# Multilevel Structural Equation Modeling of Students’ Dietary Intentions/Behaviors, BMI, and the Healthfulness of Convenience Stores

**DOI:** 10.3390/nu10111569

**Published:** 2018-10-23

**Authors:** Tanya Horacek, Elif Dede Yildirim, Kendra Kattelmann, Carol Byrd-Bredbenner, Onikia Brown, Sarah Colby, Geoffrey Greene, Sharon Hoerr, Tandalayo Kidd, Mallory Koenings, Jesse Morrell, Melissa D. Olfert, Beatrice Phillips, Karla Shelnutt, Adrienne White

**Affiliations:** 1 Department of Public Health Food Studies and Nutrition, Syracuse University, Syracuse, NY 13244, USA; 2 Human Development and Family Studies, Auburn University, Auburn, AL 36849, USA; elifdy@auburn.edu; 3 Health and Nutritional Sciences Department, South Dakota State University, Brookings, SD 57007, USA; kendra.kattelmann@sdstate.edu; 4 Department of Nutritional Sciences, Rutgers University, New Brunswick, NJ 08901, USA; bredbrenner@aesop.rutgers.edu (C.B.-B.); koenings@aesop.rutgers.edu (M.K.); 5 Department of Nutrition Science, Purdue University, Lafayette, IN 47907, USA; onb001@auburn.edu; 6 Department of Nutrition, Dietetics & Hospitality Management, Auburn University, Auburn, AL 36849, USA; 7 Department of Nutrition Science, East Carolina University, Greenville, NC 27858, USA; scolby1@utk.edu; 8 Department of Nutrition, University of Tennessee, Knoxville, TN 37996, USA; 9 Department of Nutrition and Food Sciences, University of Rhode Island, Kingston, RI 02881, USA; ggreene@uri.edu; 10 Department of Food Science and Human Nutrition, Michigan State University, East Lansing, MI 48824, USA; hoerrs@gmail.com; 11 Department of Food, Nutrition, Dietetics and Health, Kansas State University, Manhattan, KS 66506, USA; martan@k-state.edu; 12 Department of Nutritional Sciences, University of Wisconsin, Madison, WI 53706, USA; 13 Department of Molecular, Cellular & Biomedical Sciences, University of New Hampshire, Durham, NH 03824, USA; jesse.morrell@unh.edu; 14 Division of Animal & Nutritional Sciences, School of Agriculture, West Virginia University, Morgantown, WV 26506, USA; Melissa.Olfert@mail.wvu.edu; 15 Department of Food and Nutritional Sciences, Tuskegee University, AL 36088, USA; bebe62@bellsouth.net; 16 Department of Family, Youth and Community Sciences, University of Florida, Gainesville, FL 32611, USA; kpagan@ufl.edu; 17 School of Food and Agriculture, University of Maine, Orono, ME 04469-5735, USA; awhite@maine.edu

**Keywords:** young adults, consumer nutrition food environment, weight, fruit/vegetable intake, college environment, percentage k-calories from fat

## Abstract

Background: When dietary behaviors are habitual, intentions are low, and environmental cues, such as the consumer food environment, might guide behavior. How might intentions to eat healthily and ultimately actual dietary behaviors, be influenced by the consumer food environment (including the availability and affordability of healthy foods) in convenience stores? This study will determine pathways between the healthfulness of convenience stores and college students’ dietary intentions/behaviors, and body mass index (BMI). Methods: Through multilevel structural equation modeling, a comparison was made of students’ healthful meal intentions (HMI); intake (fruits/vegetables, %kcal/fat, sugar-sweetened beverages (SSBs) and whole-grains); and measured BMI; as well as the healthfulness of convenience stores (fruits/vegetables availability/quality, healthy food availability/affordability). Data was collected on 1401 students and 41 convenience stores across 13 US college campuses. Results: Controlling for gender, HMI was negatively associated with SSBs (*β* = −0.859) and %kcal/fat (*β* = −1.057) and positively with whole-grains (*β* = 0.186) and fruits/vegetables intake (*β* = 0.267); %Kcal/fat was positively (*β* = 0.098) and fruits/vegetables intake (*β* = −0.055) negatively associated with BMI. Campus level, fruits/vegetables availability were positively associated to HMI (*β* = 0.214, *β* = 0.129) and directly/negatively to BMI (*β* = −2.657, *β* = −1.124). Conclusions: HMI modifies dietary behaviors, with energy from fat and fruit/vegetable intake the most predictive of weight. Availability of fruit/vegetables in convenience stores make it easier for young adults to eat well.

## 1. Introduction

The impact of the convenience store food environment on dietary behaviors and weight status is not well studied among college students. Researchers have reported mixed results for the association between dietary behaviors and the healthfulness of foods available in stores [1,2,3,4] and restaurants [5]. In general, fruit and vegetable availability/variety/quality and price [6] and perception of availability [7] have been found to relate positively to fruit and vegetable intake. 

Various psychosocial and environment factors influence dietary behaviors and weight status. At the community food environment level (access and density), some researchers have found that access to grocery stores was correlated with dietary behaviors [8,9], but results are inconsistent [10,11,12,13]. The density of convenience stores and restaurants in a geographic area often has a negative effect on dietary quality and/or weight [10,14,15,16,17]. Many food environment researchers have used geographic information systems (GIS) analysis at an epidemiological level [11,18,19]. Fewer researchers have focused on the consumer food environment itself (e.g., availability and affordability of healthy foods) using objective environmental audits [3,20]. An assessment of the consumer food environment can potentially provide higher sensitivity to identify associations between the food environment and dietary attitudes, behaviors, and weight.

In the United States, most young adults transition to adulthood spending time on a post-secondary education college campus [21]. It is an understudied environment [22,23,24,25,26], and to date, there are limited connections discovered between campus convenience store food environment factors and young adults’ health-related attitudes and behaviors [23,24,25,27]. This environment might have a significant effect on young adults’ emerging dietary habit patterns [28,29].

Since young adults are in a transitional phase of life, they are an ideal, yet challenging sub-population for health behavior change [30]. The obesity rate for young adults has increased over the last decade from 24% to 29% [31]. Young adults’ risk for weight gain [32,33,34,35,36,37] is due in part to their poor dietary and exercise habits [36,37,38,39]. Additionally, their attitudes toward healthful eating and meal management directly influence the quality of their dietary behaviors [40].

Dietary habits have a significant influence upon the dietary quality and energy intake [41]. This influence may be mediated by dietary intentions and environmental cues, such as the availability and affordability of healthful food in the consumer food environment. However, research relating intentions to eat healthfully, the consumer food environment, and actual dietary behaviors is limited. Aggarwal and colleagues found supermarket shoppers’ attitude toward healthy eating was a key mediator of diet quality, irrespective of food costs [42]. However, due to the time pressures and transportation limitations, college students use convenience stores [27,29]. What are the associations between the consumer food environment of convenience stores and young adults’ dietary intentions and behaviors?

Bandura’s Social Cognitive Theory [43] construct of reciprocal determinism, with respect to the influence of the environment on behavior, is a useful framework for considering the relationships between the environment and college students’ attitudes and behaviors. The objective of this study was to determine the mediating factors and pathways between the components of the convenience store consumer food environment and college students’ dietary intentions, behaviors, and weight status.

## 2. Materials and Methods

This study was approved by the Institutional Review Boards of the authors’ universities (South Dakota State University, University of Maine, University of Florida, Tuskegee University, West Virginia State University, University of Wisconsin, Kansas State University, Michigan State University, University of Rhode Island, East Carolina University, University of New Hampshire, Purdue University, and Syracuse University). All participants gave informed consent.

### 2.1. Design

Bandura’s Social Cognitive Theory [43] provided the evaluative framework for the environmental, intentional, and behavioral variables selected. Bandura’s concept of reciprocal determinism—that behaviors are influenced not only by beliefs or intentions but also by the environment or external stimuli informed these theorized connections [43]. External stimuli or the environment included food store environment healthfulness and affordability; intentions included healthful meal intentions; and the influenced behaviors included fruit, vegetable, fat, and sugar-sweetened beverage intake. 

Multilevel structural equation analysis was used to determine the relationship first on an individual level, then on a campus level. On the individual level, we tested intentional/behavioral variables: students’ healthful meal intentions/behaviors, fruits/vegetables intake, % kcal from fat, sugar-sweetened beverages, and whole grains; and the direct variable: BMI; with gender as the control variable. Then, environmental variables (availability and quality of fruit and vegetables, and the availability and affordability of healthy foods) were added in for the campus level. This cross-sectional study merged two datasets: (1) Young Adults’ Eating and Active for Health (YEAH) baseline-survey conducted 2009 [44] and (2) Campus Environmental Audits for Food Stores [22] conducted 2008–2011. Data are available on request.

### 2.2. Young Adults’ Eating and Activity for the Health Sample

Data on 1401 young adults from 13 universities included dietary attitudes and intake, and weight status obtained from the YEAH baseline data [44]. A convenience sample of full-time, 18–24-year-old students were recruited (6277 responded, 3334 eligible (52%), 1639 participated in YEAH (49%); difference in this study were due to missing data). Participants were excluded from YEAH if they were an exercise, nutrition, or health major; had a body mass index (BMI) ≤18.5 kg/m^2^; or a medical diet-and/or activity-related restriction [44,45]. Data were collected using a web-based survey and in-person physical assessments.

### 2.3. Young Adults’ Eating and Activity for Health Measures

#### 2.3.1. Dietary Attitudes

Healthful mealtime intentions can be defined as the self-instruction to plan for healthful mealtime behavior (i.e., planning, choosing, and assembling healthful meals). It was measured with six items adapted [44] from interviews/focus groups conducted by Strong, et al [46]. Participants indicated the frequency over the past three months they: thought about the importance and ease of planning quick and simple meals; considered including healthy beverages and fruit/vegetables at every meal; and reminded themselves to eat in moderation and allowed room for an occasional treat. Healthful mealtime behaviors refers to self-regulation in consuming healthy snacks, beverages, and meals. It was measured using four items adapted [44] from Strong, et al. [46]. Participants indicated the frequency over the past three months they had planned healthy snacks, selected a healthy beverage, purposely choose vegetables, and been flexible/sensible with food choices. Answers for both dietary attitudes scales were assessed via a 5-point Likert scale (1 = never to 5 = always). Responses to each item were summed to create scale scores. For this sample, Cronbach alpha for the healthful meal intentions scale was 0.73 and for the meal behavior scale was 0.71. 

#### 2.3.2. Dietary Intake

The National Cancer Institute (NCI) Fruit and Vegetable Screener was used to calculate daily intake of fruit and vegetable in cups, and the NCI Fat Screener was used to determine the energy from fat as the percentage of kilocalories per day [47,48] Tested with national samples, both screeners had high correlations (0.5 to 0.8) with multiple 24-h recalls and food frequency results [47,48]. Sugar-sweetened beverage (SSB) intake, in kilocalories per day, was estimated based upon eight consumption frequency and amount questions regarding: non-diet soft drinks, fruit drinks, energy drinks, and specialty-coffee drinks; Cronbach’s alpha = 0.44 [49]. Servings of whole grains consumed on average per day were self-assessed via one question with choices ranging (<1 to ≥6). MyPlate description of whole grains and serving sizes were provided for reference [50].

#### 2.3.3. Demographics

Students also self-reported demographic data like age, gender, and race/ethnicity. Additionally, they identified their university, school year, and residence (on or off campus). 

#### 2.3.4. Anthropometrics

The dependent variable, body mass index (BMI), was calculated from measured height and weight of each participant as weight in kg divided by height in meters squared. Standard protocols were used, equipment was calibrated prior to use, and data collectors were trained and demonstrated acceptable inter-rater reliability prior to measurement [44].

### 2.4. Environmental Audit Food Store Sample

Teams at each of the 13 institutions comprising the North Central Multistate Research Project (NC1028) defined the convenience food store environment. Each team defined their environment as the campus plus a 1.5-mile radius beyond the campus geographic boundary. This definition was adopted because many students live on or near their college campus within the defined area. Then from local student input, each team selected the convenience food stores that students frequented. Convenience/drug stores (*n* = 27), and on-campus stores (*n* = 14) were audited because these venues carry food items and cater to students’ needs. Grocery stores were not included in this analysis primarily because most students (over 75%) lived on campus with limited need for, or access to transportation to, a full grocery store. Institutional Review Board approval was not necessary for these food store audits, because no human subjects were involved. All data collectors were thoroughly trained and met inter-rater reliability standards [22].

### 2.5. Environmental Audit Food Store Measures

Data collectors used a modified version of the Nutrition Environment Measures Survey for Stores NEMS-S [51] to assess the availability and quality of fruit and vegetables, and the availability and affordability of healthy foods. Interclass correlations were greater than 0.80 for all evaluators for this study [22] and NEMS-S had high test-retest reliability and was valid in varied environments [51].

The availability of fruits and vegetables separately was determined as a total count of the fresh, canned or frozen fruit or vegetables for a total possible sub-score for each of (0–24). The fresh fruits included were: bananas, apples, oranges, grapes, cantaloupe, peaches, strawberries, honeydew melon, watermelon and pears; and frozen/canned (no-added sugar) fruits were: peaches, fruit cocktail/mixed fruit, pineapple, strawberries, blueberries and raspberries. The fresh vegetables evaluated included: carrots, tomatoes, sweet peppers, broccoli, lettuce, corn, celery, cucumbers, cabbage, and cauliflower; frozen/canned (no-added sauce or fat) vegetables included: green beans, corn, carrots, peas, broccoli, spinach, and mixed vegetables. The quality of the fresh fruit and vegetables was determined based upon the percentage of the acceptable (e.g., crisp, minimal blemishes) fresh produce as compared to the total fresh produce for possible sub-scores between (0 to 6) [22].

Healthy foods comprised low-fat/lean milk, low-fat/lean or vegetarian-alternative ground beef/hot dogs, high fiber/low sugar cereal, low-fat baked-goods and healthy frozen meals, whole-grain bread and baked chips, and diet/100% juice. The presence of each healthy food category was tallied, with additional points earned for number of healthy alternatives within a food category, for a range of possible sub-scores (0 to 33). For each category, a higher score indicated more healthful and/or acceptable products. For healthy foods affordability, the original NEMS-S evaluated food categories as: (−1 = healthy more expensive than unhealthy alternative; 0 = equally priced; 1 = healthy lower price than unhealthy option). Price data were adjusted from original NEMS-S data to eliminate negative scores and associate higher score with more affordable healthy foods for a total possible range of sub-scores between (0 to 13). Average sub-scores per campus were calculated.

### 2.6. Analysis

Descriptive statistics and zero order correlations were calculated for all study variables and differences determined by gender. To account for students were nested within 13 universities, Multilevel Structural Equation Modeling with Robust Maximum Likelihood (MLR) Estimation was conducted. Mplus 8.1 (Muthen & Muthen, Los Angeles, CA, USA, 2018) software was used to assess the proposed model [52]. In the first analysis, individual level variables were entered in to the model to assess the direct and indirect associations between healthful meal intentions, energy from fat, SSB Kcal, whole grain intake, total fruit and vegetable intake, and students’ body mass index. Energy from fat, servings of whole grains per day, and daily fruit and vegetable intake in cups were allowed to intercorrelate in the model. Gender was included as a control variable. In the second model, campus level variables (availability and quality of fruit and vegetables, and the availability and affordability of healthy foods) were ground mean centered and entered in to the model. 

Missing data less than 5% were addressed using Full information maximum likelihood method (FIML). A probability level of 0.05 was used for statistical significance. The overall goodness of fit for the within-level and multilevel effects were assessed by the Chi-square statistic, degrees of freedom (df), Comparative Fit Index (CFI) [53], Root-Mean-Square Error of Approximation (RMSEA) [54], and Standardized Root Mean Square Residual (SRMR). A Chi-square with *p*-value less than 0.05 suggested goodness fit, as did CFI greater than 0.95, and RMSEA less than 0.05, and SRMR values less than 0.10 indicated good fit for the model [54].

## 3. Results

The majority of the student sample (*n* = 1401) were white (65%), female (66%), of “normal” weight status (67.9%), first- or second-year students (73.2%), who lived on campus (96.8%) and with a mean age of 19.33 ± 1.07 SD years. Demographic characteristics of the sample population and the convenience stores are in Table 1.

The zero-order correlations of model variables were shown in Table 2. Most variables differed significantly by gender and energy from fat and whole grain intake, as shown in Table 3. Men had higher BMI (*p* < 0.05), got more energy from SSB (*p* < 0.001), and had higher fruit and vegetable intake (*p* < 0.05), while women had higher scores for healthful meal intention (*p* < 0.001), indicating a need to use gender as a control variable in the model.

### 3.1. Individual Level Analysis

**Figure 1:**
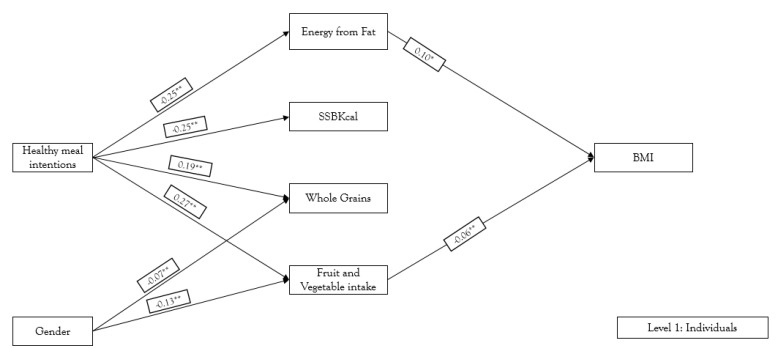
Multilevel structural equation modeling: Level 1—individual intentions/behaviors. * *p* < 0.05; ** *p* < 0.001 Definitions: Healthy meal intentions: self-instruction to plan for healthful mealtime behavior (i.e., planning, choosing, and assembling healthful meals); Energy from Fat: % Kcal/Fat/day; SSB Kcal: Sugar-sweetened beverages intake/day; Whole Grain: Whole grain intake/day, Fruit and vegetable intake—intake per day as determined by NCI food frequency; BMI: body mass index, calculated from assessed weight and height.

We first assessed the associations between healthful meal intentions, energy from fat, SSB kcal, whole grain intake, total fruit and vegetable intake, and BMI. The model fit indicated a good fit to data: chi square (*n* = 1401, df = 4) = 7.09, *p* < 0.05, CFI = 0.992, RMSEA = 0.023, and SRMR = 0.018. The direct and indirect associations shown in Figure 1 can be summarized as follows. After controlling for gender, healthful meal intentions were negatively associated with energy from fat (*β* = −0.251, *p* < 0.001), and SSB kcal (*β* = −0.249, *p* < 0.001), and positively associated with whole grain intake (*β* = 0.186, *p* < 0.001), and total fruit and vegetable intake (*β* = 0.267, *p* < 0.001). There were direct positive associations between energy from fat and BMI (*β* = 0.098, *p* < 0.05), and negative associations between total fruit and vegetable intake and BMI (*β* = −0.055, *p* < 0.001). Healthful meal intentions was indirectly associated with BMI via energy from fat (*β* = −0.025, *p* < 0.10), and total fruit and vegetable intake (*β* = −0.015, *p* < 0.001).

### 3.2. Campus Level Analysis

Next, we assessed the relationships between campus level environmental variables (availability and quality of fruit and vegetables, and the availability and affordability of healthy foods) and student level mediating or intentional/behavioral variables: students’ healthful meal intentions, fruits/vegetables intake, energy from fat, SSB kcal, and whole grains; and the direct variable, BMI. The model fit indicated an acceptable fit to data: chi-square (*n* = 1401, df = 34) = 84.527, *p* < 0.001, CFI = 0.909, RMSEA = 0.083, and SRMR = 0.019.

**Figure 2:**
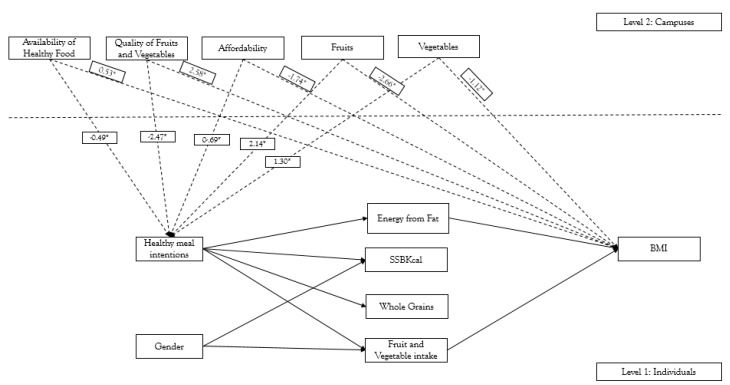
Multilevel structural equation modeling: Level 2—Campuses healthfulness of store environment and BMI. * *p* < 0.05; Definitions: Availability of Healthy Food: The total of 13 food categories from NEMS-S, Quality of Fruits and Vegetables: for fresh produce; Affordability: healthy food affordability; Fruits: Availability of fruits (fresh, frozen and canned); Vegetables: Availability of vegetables (fresh, frozen and canned); Healthy meal intentions: self-instruction to plan for healthful mealtime behavior (i.e., planning, choosing, and assembling healthful meals); Energy from fat: % Kcal/Fat/day; SSB Kcal: Sugar-sweetened beverages intake/day; Whole Grains: Whole grain intake/day, Fruit and vegetable intake: intake per day as determined by NCI food frequency; BMI: Body Mass Index—calculated from assessed weight and height.

There was a direct positive association between the availability of fruit (*β* = 2.140 *p* < 0.05) and availability of vegetable (*β* = 1.294, *p* < 0.05) and healthful meal intention indicating campuses which had fruit and vegetable availability above the ground mean predicted higher levels of healthful mean intentions (Figure 2). However, there were negative associations between quality of fruit and vegetables (*β* = −2.467, *p* < 0.05), the availability (*β* = −0.490, *p* < 0.05) and affordability of healthy foods (*β* = 0.693, *p* < 0.05) and healthful meal intention suggesting that college campuses which had quality of fruit and vegetables, the availability and affordability of healthy foods below the ground mean had higher levels of healthful meal intentions. Across campuses, higher levels of availability of healthy foods were associated with lower levels of energy from fat (*β* = −0.846, *p* < 0.001).

Campus level healthy meal intentions were negatively associated with average individuals’ SSB Kcal (*β* = −0.859, *p* < 0.001) and energy from fat (*β* = −1.057, *p* < 0.001). BMI was lower among campuses which had higher scores of affordability of healthy foods (*β* = −1.742, *p* < 0.05), availability of fruit (*β* = −2.657, *p* < 0.05), and availability of vegetable (*β* = −1.124, *p* < 0.05), whereas BMI was higher among campuses which had higher scores of availability of healthy foods (*β* = 0.531, *p* < 0.05) and quality of fruit and vegetables (*β* = 2.581, *p* < 0.05).

## 4. Discussion

To our knowledge, this is the first report of an investigation of mediators between (1) convenience store consumer environment, (2) meal intentions, (3) dietary intake, and (4) weight status for college students. Using multilevel structural equation modeling, this study tested the relationships as predicted by the Social Cognitive Theory [43] on an individual, and then a campus level. First on an individual level, this study determined that although there were logical links between student’s healthy meal intentions and the four dietary behaviors (increased whole grains and fruit and vegetable; decreased energy from fat and sugar-sweetened beverages intakes), only decreased energy from fat and increased fruit and vegetable intakes related to decreased BMI. Then for the campus level, there were mixed results. Higher fruit and vegetable availability and affordability of healthy foods in convenience stores for a campus related to decreased BMI. Whereas, increased quality of fruits and vegetables and availability of healthy foods for a campus related to increased BMI. As for the relationship between convenience store sub-scores and students’ healthy meal intentions: as the availability of fruits and vegetables increased for a campus, so too did the students’ healthy meal intentions; whereas there was a reverse relationship with quality of fruits and vegetables; affordability and availability of healthy foods, as they were negatively related to healthy meal intentions.

On an individual level, healthful meal intentions were positively related to whole grains and fruit and vegetable intake, and negatively associated with energy from fat and SSB intake. Energy from fat and decreased fruits and vegetables were predictive of BMI. McDermott and colleagues found that age, but not gender, was a modifier of the association between intentions and dietary behaviors [40]. In contrast, for the current study, with a narrow age range, we found that it was necessary to control for gender. Women had higher intakes of whole grain and fruit and vegetables than men. The effect of gender in the current study; is consistent with the findings of other studies [25,38,55].

On the campus level, using the Social Cognitive Theory [43], environmental variables such as, availability of fruits and vegetables in convenience stores influenced BMI mediated by healthful meal intentions and the dietary behaviors energy from fat and SSB intake. Quality of fruits and vegetables, and availability and affordability of healthy foods were negatively associated with healthful meal intentions. Availability of healthy foods was also negatively associated with energy from fat. Environmental variables were also directly related to BMI: affordability of healthy foods, and availability of fruit and vegetables were negative associated with BMI, while quality of fruits and vegetables and availability of healthy foods were positively associated with BMI.

The mediation by healthful meal intentions between environmental and behavioral variables was another important finding of the current study. These findings are consistent with findings from two recent meta-analyses [40,56] of the strength of association between intentions and behaviors. Adriaanse and colleagues [56] found a positive association between effective implementation intentions and healthy eating behaviors with an overall medium effect size. A meta-analysis using the Theory of Planned Behavior [40] found intentions were related to health-enhancing dietary behaviors and to food avoidance behaviors.

Most researchers who have evaluated the effect of food environment on weight have utilized an epidemiological approach, using GIS and store density [57]. Researchers have focused upon neighborhoods in urban or suburban settings regarding the effect of the consumer food environment on health habits and weight [1,13,58]. Gustafson, et al. [13] found a non-significant association, between the availability of healthy foods and BMI for a group of North Carolina women, and Cerin et al. [1] found no association between healthy food availability and BMI for higher-income neighborhoods in Atlanta. On the other hand, Casagrande, et al. [58] found a positive association between healthy food availability and higher BMI among individuals living in primarily white neighborhoods near Baltimore. Like Casagrande et al. [58], in the current study of college students from multiple areas of the north and southeast, there was a direct association between BMI and healthy food availability and also the quality of fruits and vegetables. 

When considering the associations between healthful food in small stores and food intake, it is hard to draw conclusions from the diverse results reported in the literature. In the current study, at the campus level, although fruit availability and vegetable availability (mediated by healthy meal intentions) were each inversely associated with students’ energy from fat and SSB intake, they were not related to fruit and vegetable intake. Similarly, researchers in New York City reported that the availability of fruits and vegetables in convenience stores were independently and inversely related to SSB purchases [59]. In contrast, Bodor et al. [6] found a positive association between vegetable availability and vegetable intake, but no association between fruit availability and fruit intake in their study in central-city New Orleans. As previously mentioned, Gustafson et al. [13] found a trend between the availability of healthy foods and fruit and vegetable intake. While healthful food accessibility seems to be associated with diet quality, there is not strong evidence from these studies that accessibility is related to fruit and vegetable intake. Although fruit and vegetable intake and decreased energy from fat were predictive of BMI at an individual level, the effect of the environment had more influence on energy from fat in this study. Therefore, more than just having fruits and vegetables available in small stores may be necessary to affect intake. For example, using persuasive appeals to direct students’ intentions [60], such as marketing fruits and vegetables and locating them at the front of the store [59] or point of purchase [61] has been found to influence purchases and consumption.

The influence of healthy food affordability in this model was also interesting. The affordability of healthy foods was negative related to both healthy meal intentions, energy from fat and BMI. So as healthy food was more affordable, energy from fat and BMI decreased but so did the students’ healthy meal intentions. It is possible that when healthy food is more affordable, students do not have to focus as highly on their meal intentions to meet their goals. Powell et al. [62] found that the price of fruit and vegetables was inversely related to fruit and vegetable intake for young adults. In comparison, other consumer food environment researchers have found no relationship between healthy food affordability and intake [9,63].

For this study, on the campus level, the convenience store consumer environment variables of healthy foods availability and quality of fruit and vegetables did not meaningfully relate to students’ behaviors. However, on the individual level, healthful meal intentions meaningfully related to intake, positively with whole grains, fruits and vegetables and negatively with percentage of calories from fat and SSB intake. Similarly, Graham et al. [64] found that as Minnesota college students perceived more access barriers (including quality and affordability) to fruits and vegetables, but less perceived personal barriers (skills, preference), they had higher consumption of fruits and vegetables. These negative associations found in both studies regarding access indicates that those who intended to eat healthy were willing to work harder to find healthy foods when the quality of fruits and vegetables were lower. 

A key implication of the fact that healthful meal intentions was the main mediator between convenience store consumer environment and college students’ dietary behaviors is that healthful meal intentions should be further assessed and may be an important target for driving improvements in their dietary behaviors. Kattelmann et al. [44] found that healthful meal intentions were positively influenced by a dietary intervention with young adults; however, Kothe and Mullan [65] found that changes in implementation intentions for a sample of young adults did not correlate to their fruit and vegetable consumption. Based on these mixed results, more research is necessary to determine the ability to influence healthful meal intentions and their role in facilitating healthy dietary behaviors. If healthful meal intentions can be influenced and do facilitate healthy dietary behaviors, then they would be central to developing effective intervention and environmental supports.

Convenience stores consistently have been found to be less healthy than grocery stores [2,66]. While they are a potential avenue for affecting dietary change [59,67,68,69], numerous researchers have identified barriers to making healthy choices in these stores [22]. The barriers include low availability of fruits and vegetables (35–50%), healthy snacks, or healthy staple items [70,71]. In a large national evaluation, small stores had only 0.60 relative availability of healthier food alternatives. Over 82% of convenience type stores carry every category of SSB, while only 36–42% carry 1%-skim milk [72]. Young adults lack the motivation to eat fruit/vegetables as evidenced by their limited intake [73,74], researchers need to advocate for changes in the environment/food industry, particularly convenience stores to help young adults’ make “the healthy choice, the easy choice” [75,76]. 

Strengths of this study included a large sample size of young adults, objective measurement of weight and height to determine BMI, and the use of valid and reliable questionnaires. In addition, researchers objectively measured the healthfulness of the stores via the audit of the consumer food environment. 

### Limitations

This study presents associations, not causation, and was based upon self-reported dietary intake data and had a small effect size *β* < 0.30. This small effect size may be related to the study focus on convenience stores on or around campus. However, because of the importance of convenience stores to college students and the lack of research relating to convenience stores, the study finding of an association is an important contribution to the literature. In addition, unequal cluster sizes and the small number of clusters limit the generalizability of the results. Future research regarding this type of structural equation modeling analysis should be expanded to include other psycho-social factors which have been found to affect health behaviors, such as perceptions of the environment and social support [64,76]. Perception should be included particularly since some researchers have found subjects’ perception of the environment to relate to behavior [13,77,78] while others have only found a weak association [79]. Assessing a mix of community and consumer food environment variables together with dietary and psycho-social variables should provide an improved comprehensive model with better predictive power [5,25,78].

## 5. Conclusions

Young adults in college are at a pivotal point to determine their long-term health habits for decreasing their risk for chronic diseases and obesity. Too many interventions focus only on the behaviors students need to change. This study provides insight into the association between the convenience store consumer food environment, healthy eating, and weight for a population of college students. These findings can direct policies and interventions for healthful weight management and health promotion on college campuses. Specifically, making fruits, vegetables, and healthy foods affordable in small convenience stores on and near college campuses may positively affect students weight status. Additionally, environmental supports and policy changes that encourage and make the healthy choice the easier choice (marketing, placement, tastings, healthy snack deals) [59,60,75,76] at convenience stores need to be tested to determine their ability to increase college students’ dietary quality. To compliment environmental interventions and supports, researchers have suggested that healthful meal intentions are subject to change through intervention [44]. With this study, we found they were an important mediator between the environment and the students’ actual dietary behaviors, therefore may be an important target for interventions guiding college students to make healthy dietary choices.

## Figures and Tables

**Table 1:** Mean and frequencies of demographic variables for college students (*N* = 1401) and convenience stores (*n* = 41). BMI: body mass index.

Variable	Statistics % (*n*)	*χ* ^2^	*p*
Age	Mean ± SD (19.33 ± 1.07)		
BMI category		803.77 (df = 2)	<0.001
Normal weight	67.9% (951)		
Overweight	23.6% (330)		
Obese	8.4% (118)		
Gender		141.34 (df = 1)	<0.001
Male	34.1% (478)		
School Year		935.58 (df = 4)	<0.001
Freshman	38.7% (542)		
Sophomore	34.5% (484)		
Junior	23.9% (335)		
Senior	1.3% (18)		
Residence		2471.54 (df = 5)	<0.001
On campus residence hall	63.4% (888)		
On campus sorority or fraternity	3.8% (53)		
On campus-other college housing	7.6% (107)		
Off campus housing	20.6% (289)		
Off campus parent/guardian’s home	2.4% (34)		
Off campus -other	0.8% (11)		
Race		2777.61 (df = 5)	<0.001
White	64.6% (905)		
Black or African American	12.9% (181)		
Asian	9.0% (126)		
American Indian or Alaska Native	0.8% (11)		
Native Hawaiian or Other Pacific Islander	0.5% (7)		
Other	4.0% (56)		
Ethnicity			
Hispanic	5.7% (80)		
**Convenience Store Variables**	**Mean ± SD**		
Healthy food affordability	8.11 ± 0.09		
Healthy food availability	9.75 ± 2.91		
Fruit/vegetable quality	2.19 ± 1.85		
Fruit availability	1.63 ± 0.64		
Vegetable availability	1.05 ± 0.65		

**Table 2:** Zero-order correlations and means for all study variables (*n* = 1401).

Individual Variables	1.	2.	3.	4.	5.	6.	7.	8.	Mean	± SD
1. Gender	1.0									
2. BMI	−0.059 **	1.0							24.13	4.31
3. Healthful meal intentions	0.212 **	−0.043	1.0						3.19	0.77
4. Healthful meal behaviors	0.145 **	−0.127 **	0.681 **	1.0					3.32	0.77
5. % kcal/ Fat/day	−0.019	0.125 **	−0.244 **	−0.300 **	1.0				31.18	5.06
6. SSB kcal/day	−0.134 **	0.054 *	−0.263 **	−0.284 **	0.300 **	1.0			157.45	243.28
7. Whole grain/day	−0.026	−0.037	0.171 **	0.231 **	−0.115 **	−0.101 **	1.0		2.07	1.48
8. F/V intake/day	−0.075 *	−0.065 *	0.2420 **	0.373 **	−0.098 **	−0.003	0.168 **	1.0	2.72	2.33

BMI: body mass index, Healthful meal intentions: self-instruction to plan for healthful mealtime behavior (i.e., planning, choosing, and assembling healthful meals); Healthful meal behaviors: the self-regulation for consuming healthy snacks, beverages, and meals; % kcal/fat/day: energy from fat intake, SSB kcal/day: sugar-sweetened beverages intake, whole grain/day: whole grain intake, F/V intake/day: fruit and vegetable intake. * *p* < 0.05. ** *p* < 0.001.

**Table 3:** Differences by gender for key variables (*n* = 1401).

Individual Variables	Male		Female		*t*-Value (df = 1399)	*p*
	Mean	± SD	Mean	± SD		
BMI	24.48	± 3.97	23.95	± 4.47	2.273	<0.05
Healthful meal intentions	2.97	± 0.82	3.31	± 0.72	−7.672	<0.001
Healthful meal behaviors	3.17	± 0.79	3.40	± 0.74	−5.300	<0.001
% kcal/fat/day	31.31	± 4.74	31.11	± 5.22	0.686	NS
SSB/kcal/day	202.90	± 276.74	134.21	± 220.84	4.614	<0.001
Whole grain servings/day	2.12	± 2.12	2.04	± 1.44	0.926	NS
Fruit and vegetable intake cups/day	2.97	± 2.97	2.60	± 2.27	2.651	<0.05

BMI: Body mass index, calculated from assessed weight and height; Healthful meal intentions: self-instruction to plan for healthful mealtime behavior (i.e., planning, choosing, and assembling healthful meals); Healthful meal behaviors: the self-regulation for consuming healthy snacks, beverages, and meals; % kcal/fat/day = energy from fat intake; SSB kcal: sugar-sweetened beverages intake/day; whole grain servings/day = daily servings consumed of whole grains; fruit and vegetable intake cups/day: daily intake of fruits and vegetables as determined by NCI food frequency.
